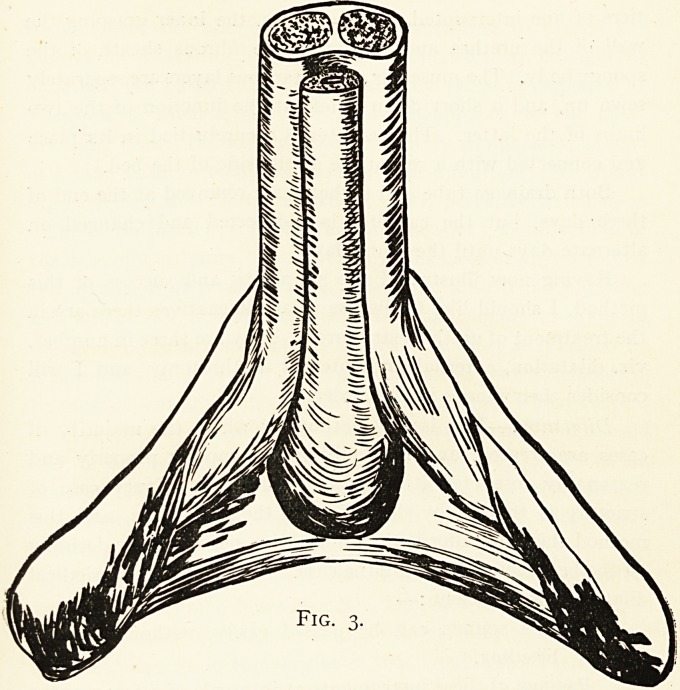# On the Excision of Strictures of the Urethra

**Published:** 1910-12

**Authors:** Ernest W. Hey Groves

**Affiliations:** Assistant Surgeon to the Bristol General Hospital.


### ON THE EXCISION OF STRICTURES OF THE URETHRA.

BY

Ernest W. Hey Groves, M.S., F.R.C.S.,

*Assistant Surgeon to the Bristol General Hospital.*

---

It is somewhat remarkable that in these days, when much ingenuity and daring are expended upon new operative procedures, so little attention has been paid to the radical atretment of urethral strictures.

It is true that most modern text-books do mention the excision of strictures in a brief and unconvincing sort of way, after they have devoted many pages and illustrations to the detailed description of the various forms of urethrotomy. Isolated papers appear from time to time dealing with this subject; but nevertheless the radical operation for urethral stricture is not as widely known, or practised, as it well deserves to be. It is not taught as a routine method of operative surgery. It is owing partly, I suppose, to the zeal of instrument makers, partly to the fascination that especially elaborate instruments have for some people, and partly to the important part that these instruments are made to play in students' examinations, that urethrotomy holds its place as the principal operation for severe urethral strictures.

In this respect the urethrotome has played the same part in hindering the proper radical treatment of urethral stricture as has the guillotine in that of tonsillar enlargement.

I would therefore venture to bring forward the results I have obtained in a small series of cases of this operation, in order that I may have the opportunity of describing the details of the method, and pointing out the great advantages that it presents over dilatation and urethrotomy.

During the last seven years I have had six cases of this operation, and although I am aware that this is not a sufficient number on which to base any very far-reaching conclusions, yet as the results obtained have been so uniformly good I think that they may be fairly taken in conjunction with the many cases published in recent surgical literature as illustrating the advantages of the method. I have had no fatality, no untoward complication, and as far as I have been able to ascertain, permanent success in all. A short history of three of my cases will suffice to exemplify the method and its results.

**Case 1.**—F. B., a railway porter, aged 63, had been suffering for about seven or eight years from difficulty in micturition. He alleged that this was the result of a strain, but there was no confirmation of this, and the stricture was probably inflammatory. Only the smallest instruments could be passed (No. 4), and these caused pain and bleeding. *Operation*, April 19th, 1908. Excision of a stricture about one inch long, situated just in front of the triangular ligament. He left the hospital within a fortnight, and he neglected to attend as an out-patient, and he afterwards explained that this was because he had no further difficulty in micturition. Two years after the operation he began to find it necessary to strain in passing water. For this reason he came to see me in September, 1910. At first only a small bougie could be passed, but after this had been done, there was not the slightest difficulty in passing others up to No. 12 (Eng. size). So that within two and a half years of the operation he has only required the use of instruments once.

**Case 2.**—J. P., a labourer, aged 27. Nine months previously he had sustained a severe accident, by which he ruptured the urethra. A supra-pubic cystotomy was done at the time by Dr. Toye of Bideford, who was unable to introduce any instrument into the bladder by the urethra. By this means extravasation was prevented, but nevertheless some leaking and suppuration occurred in the perineum, and when this was opened, urine escaped from the wound on the inner side of the right thigh. It required three months for the supra-pubic and perineal wounds to heal. For the next six months he suffered from dribbling and incontinence, but no instrument could be passed into the bladder. Dr. Toye sent him to me, and I found that I could only pass a small sound into the urethra, where it became so tightly gripped by the dense stricture in the region of the triangular ligament that it could not be passed into the bladder. It seemed as hopeless a case as one could have, and accordingly I gave the patient a very guarded prognosis. At first the operation was one of extreme difficulty, owing to the fact that nothing could be passed through the stricture. The whole region of the triangular ligament and membranous urethra was occupied by a dense mass of scar tissue, but at length, after cutting through this in the mid line, the urethra proximal to the stricture was opened, and was found to be both dilated and hypertrophied. This circumstance made the rest of the operation quite easy, for although the stricture was so impenetrable that no channel could be found through it, yet was only about half an inch long, and the large and thick urethra was well adapted for suturing. He made a speedy recovery, and I have recently heard from Dr. Toye that two months after the operation a full-sized instrument could be easily passed, and that he can deliver himself of a full and ready stream of urine.

**Case 3.**—W. T., a workman, aged 48. Admitted into the Cossham Hospital, October, 1910. He had had an attack of gonorrhœa three years ago, and for nearly two years he has had difficulty in micturition. No instrument could be passed, the arrest occurring about five inches from the meatus. The operation was easier than either of those just described, because the stricture was well in front of the triangular ligament. It was less than one inch long, and the impossibility of passing any instrument was due to the existence of a false passage. Before he left the hospital a full-sized rubber catheter could be readily passed into the bladder.

These three cases are fairly representative of impassable strictures of the urethra. The first was the usual inflammatory contraction just at the bulb, the second was traumatic, following a complete rupture, whilst the third was a penile stricture.

As I have said in my introductory remarks, the technic of the operation of excision of urethral strictures is not described at all in current text-books. It will be worth while, therefore, to discuss the operative details in their bearing upon the feasibility and success of this treatment.

No special preparation is necessary other than that which is usual for ordinary perineal operations. The first debatable point concerns the question of the necessity for a supra-pubic opening of the bladder. Thomson Walker[1] strongly advises it, urging that the urethral wound will heal much more readily if the urine is diverted from its course. Although I have never adopted this plan, I am very favourably inclined towards it, and I shall always use it in future in cases with cystitis, and also in every case in which an instrument cannot be passed

[1] Thomson Walker in *Burghard's System of Operative Surgery*, vol. iii.

FIG. I.

A. *Internal Pudic Artery.*

through the stricture. In this way the very great difficulty of finding the proximal end of the urethra without the guide of a sound can be readily overcome by passing a full-sized instrument through the internal meatus by way of the upper opening in the bladder.

The patient is placed in the lithotomy position, and a metal bougie held in the urethra and kept accurately in the middle line of the body. A median incision is made in the perineum which stops short one inch in front of the anus. A curved transverse cut is then made almost from one ischial tuberosity to the other, coming forward to meet the posterior end of the first incision. This ⊥ shaped opening allows three flaps of skin and fascia to be turned back. In the area thus exposed are seen the ejaculator urinæ muscle in front and the transversus perinei behind (Fig. 1), the former covering the corpus spongiosum and the latter marking the posterior limits of the dense, triangular ligament. The ejaculator muscle is divided in the mid line and its two halves turned outwards. In recommending this as a separate step of the operation I have the following reasons: the muscle can subsequently be united as a distinct layer over the seat of anastomosis, and the urethra will be more accurately exposed if the layers covering it are divided one by one, just as the trachea is, in the operation of tracheotomy.

Now comes the cardinal point in the operation, the scientific reason for which was first pointed out by Goldmann.[1] *The excision, suturing, and anastomosis must involve the whole thickness of the spongy body,* and not consist in an attempt to deal with the urethra inside the tissues of the corpus spongiosum. The advantages of this are obvious, because whilst it is impossible to mobilise the urethra inside the spongy body to more than a very slight extent, the corpus spongiosum itself can be mobilised to almost any extent. In Fig. 2 is represented in a somewhat diagrammatic manner the root of the penis after the muscles have been dissected off it. It illustrates clearly how the spongy body lies in a kind of trough formed by the corpora

[1] Goldmann, *Lancet*, 1906, i, 20.

cavernosa, thus making it possible to isolate as much of the former as may be necessary. The objection to thus dealing with the whole thickness of the spongy body and not merely with the urethra has been a fallacious one, founded on an imperfect knowledge of the anatomy of the vascular supply of these parts. In Fig. 1 is shown the main arteries to the penis. Piercing the base of the triangular ligament, the internal pudic artery divides into two main branches, one to the bulb and spongy body the other to the cavernous body and dorsum of

FIG. 2.

the penis. But Prof. Goldmann has demonstrated that the last-named vessels not only supply the skin of the penis, but their largest branches end in the tissues of the glans, which after all is merely the expanded end of the spongy body. Fig. 3 demonstrates this fact in an ingenious manner. It was obtained by injecting a bismuth mixture into the dorsal artery of the penis, and taking a skiagram of the organ. It will be observed that the whole substance of the bulb and spongy body have been filled by the injection. By this mechanism it is provided that the spongy body is richly supplied with blood by vessels which enter it at both ends, so that it may be freely divided, and a section of any length removed without endangering its nutrition. In fact, the only limit to the length of spongy body and urethra which can be removed is the disproportion produced between the lengths of the spongy and cavernous bodies. If this is too great, a downward bend will be given to the penis when in a state of erection. Goldmann noted this occurrence after excising a stricture $3\frac{1}{4}$ in. long, and I have experienced it

FIG. 3.

in a case of about $1\frac{1}{2}$ in. But as all the tissues of the penis are eminently elastic, this matter very soon rights itself.

The great majority of strictures lie close against the triangular ligament, this being determined by the facts that the urethra becomes torn at the most fixed point, and that the most intractable ulceration occurs in the part which is most dependent, where the infective material stagnates. Cock first pointed out, in describing his classical operation for retention of urine, that one can always rely upon finding the urethra deep to the triangular ligament dilated and thickened. This part is defined, and after the stricture has been excised it is united to the corpus spongiosum in front, the latter having been freely raised from its bed. The union is effected over a rubber catheter by two tiers of fine interrupted catgut sutures, the inner grasping the wall of the urethra and the outer the fibrous sheath of the spongy body. The muscular and cutaneous layers are separately sewn up, and a short drain is left at the junction of the two limbs of the latter. The catheter is securely tied in its place and connected with a receptacle at the side of the bed.

Both drainage tube and catheter are removed at the end of three days, but the catheter is re-inserted and changed on alternate days until the tenth day.

Having now illustrated the simplicity and success of this method, I should like to discuss what alternatives there are in the treatment of urethral strictures. They are three in number, viz. dilatation, external and internal urethrotomy, and I will consider their merits in this order.

*Dilatation.*—This is the method by which the majority of cases are treated, and no doubt if its scope is properly and reasonably restricted it is quite satisfactory. Every case of stricture is treated by dilatation in the first place, and this method therefore affords the information necessary for deciding on the best means of curing the case. Cases in which periodical dilatation is sufficient :—

Lister's sounds can be passed easily, without pain or bleeding.

Passage of these instruments at intervals of a few months is sufficient to prevent contraction.

The patient being satisfied with this method.

This is, I think, eminently a question which the patient himself ought to decide, whether he shall resign himself to dilatation for the rest of his life, or be radically relieved of his stricture. I would never persuade a patient who is satisfied with dilatation to submit to operation, but I think it is our duty to inform him that his condition could be cured. The position is analagous to that of a patient who is wearing a truss for a hernia. But it is a very different matter in the case of a man who has a stricture which can only be entered by the exercise of patient ingenuity, in whom the stricture rapidly contracts to a small size, rendering him liable to retention at any minute, and in one who, from his social status or calling, is unable or unwilling to attend regularly for dilatation. In such conditions we have no right to endanger the patient's life and health by pursuing a policy of *laissez-faire,* when a safe and ready means lies to our hand of curing him. Here the position is strictly comparable to that of a man with an enlarged prostate and retention. We might argue in this case that the patient could perfectly well pass a catheter for himself, a proceeding much easier generally than in the case of a stricture. And yet surgeons have eagerly adopted the operation of prostatectomy chiefly because it is something new and showy, and because its author says wonderful things in its favour. Now the mortality of prostatectomy as revealed by hospital statistics is over forty per cent., and that admitted by its most ardent advocates is eight to ten per cent., whilst there is at present no recorded death from urethral excision ! I should like to ask whether any surgeon can point to a series of six consecutive cases of prostatectomy being the first which he has ever done without a death, and with uniformly satisfactory results ?

*Internal Urethrotomy.*—I can dismiss the other operative procedures in much fewer words. They are merely relics of an age when every operation was done in fear and trembling, and the ideal was to do the very least that would afford temporary relief. Internal urethrotomy can only be practised for strictures which admit a No. 4 sized bougie. It only makes a nick in the scar tissue of the stricture, which permits dilatation to be then pursued in its wearisome and inconclusive methods. It is utterly useless in cartilaginous strictures and in any case which is not followed up by regular dilatation.

*External Urethrotomy.*—If this operation is reserved as one of emergency, in cases of acute retention of urine, then there is something to be said in its favour. But if we consider the matter without the bias of the text-book teaching of twenty years ago, even as an emergency expedient, it ought to be abandoned in favour of supra-pubic cystotomy, which can be done in a few moments under a local anæsthetic, and followed up later on, if the patient's condition permits, by a radical operation. But for external urethrotomy as an operation intended to cure a stricture nothing favourable can be said. What would be thought of a surgeon who was content to leave a patient with a colostomy, when by an extra-peritoneal operation he could remove the intestinal obstruction ? Like the other form of urethrotomy, it is useless unless followed up by regular dilatation. In twelve cases reported by Thomson Walker nine required the operation to be repeated, some of them more than once. For the performance of external urethrotomy the most difficult part of the operation of excision has to be carried out, and yet it stops short of the removal of the fons et origo mali, even at the moment when this lies under the very hand of the surgeon.

I believe that it is only because the anatomical possibility and the clinical success of the excision of urethral strictures is not yet realised that it has not yet been adopted as the routine treatment of all cases not suitable for dilatation

REFERENCES.

Watson and Cunningham, *Diseases and Surgery of the Genito-urinary System.* (In this a collection of 64 cases of excision is made.)
Berg, *Ann. Surg.*, 1903, xxxvii, 486.
Thomas, *Brit. M. J.*, 1902, ii, 1528.
Rutherfurd, *Lancet*, 1904, ii, 751.

## Figures and Tables

**Fig. 1. f1:**
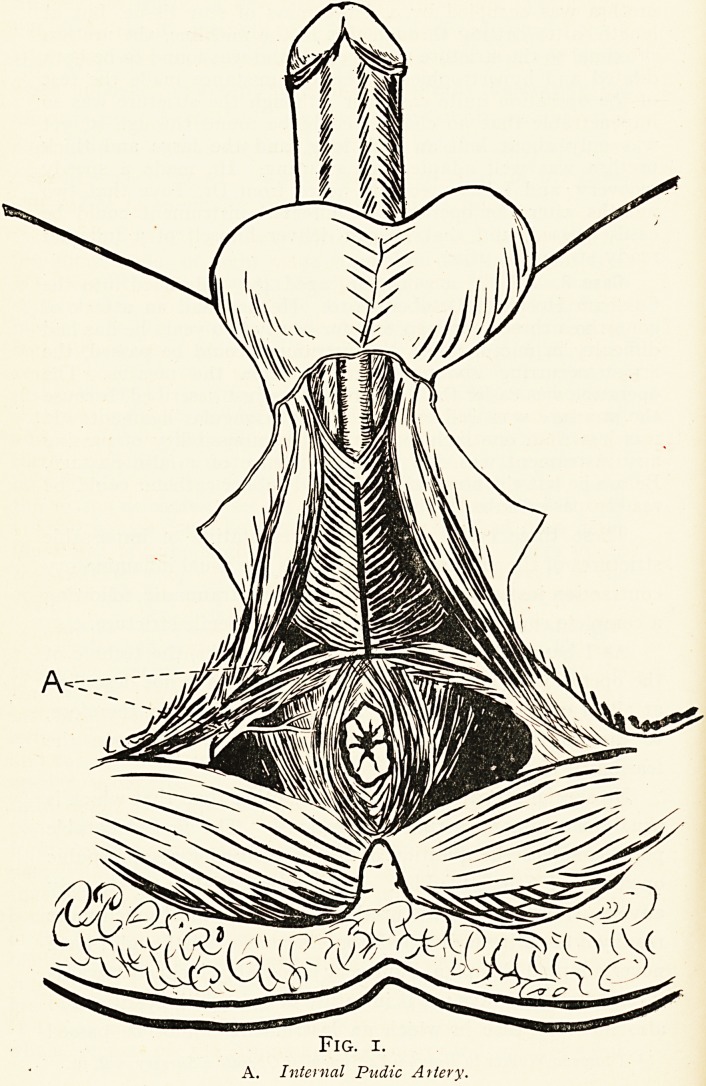


**Fig. 2. f2:**
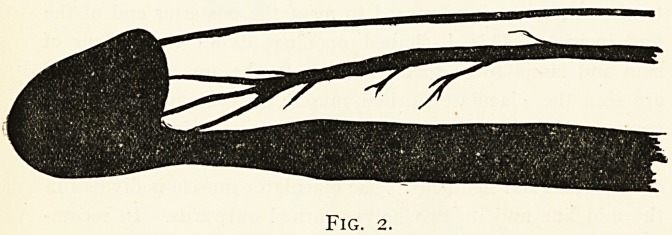


**Fig. 3. f3:**